# HPV genotypes detected in cervical cancers from Alaska Native women, 1980–2007

**DOI:** 10.3402/ijch.v72i0.21115

**Published:** 2013-08-05

**Authors:** Janet J. Kelly, Elizabeth R. Unger, Eileen F. Dunne, Neil J. Murphy, James Tiesinga, Kathy R. Koller, Amy Swango-Wilson, Dino Philemonof, Xay Lounmala, Lauri E. Markowitz, Martin Steinau, Thomas Hennessy

**Affiliations:** 1 Alaska Native Tribal Health Consortium, Anchorage, AK, USA; 2 Centers for Disease Control, Atlanta, GA, USA; 3 Centers for Disease Control, Anchorage, AK, USA

**Keywords:** Alaska Native, HPV, cervical cancer, HPV genotypes

## Abstract

**Background:**

Human papillomavirus (HPV) vaccine prevents cervical pre-cancers and cancers caused by HPV types 16 and 18. This study provides information on the HPV types detected in cervical cancers of Alaska Native (AN) women.

**Methods:**

Cases of invasive cervical cancer diagnosed in AN women aged 18 and above between 1980 and 2007 were identified from the Alaska Native Tumor Registry. A representative formalin-fixed, paraffin-embedded archived pathology block was retrieved and serially sectioned to allow histologic confirmation of lesion (first and last sections) and PCR testing of intervening sections. Extracted DNA was tested for HPV using Linear Array HPV Genotyping Test (Roche Diagnostics) with additional INNO-LiPA HPV Genotyping Assay (Innogenetics) testing on negative or inadequate specimens. All specimens were tested for a minimum 37 HPV types.

**Results:**

Of 62 cervical cancer specimens evaluated, 57 (91.9%) contained one or more HPV types. Thirty-eight (61.2%) cancers contained HPV types 16 or 18, and 18 (29%) contained an oncogenic type other than type 16 or 18.

**Conclusions:**

Overall, almost two-thirds (61.2%) of the archived cervical cancers had detectible HPV types 16 or 18, a finding similar to studies of US women. As expected, a proportion of cancers would not be prevented by the current vaccines. HPV vaccination and cervical cancer screening are important prevention strategies for AN women.

There are an estimated 11,000 cervical cancers each year in the United States; the cervical cancer burden among Alaska Native (AN) women is 10.3 per 100,000 and ranks 8th in the number of cancer diagnosed in AN women. Cervical cancer rates were *four* times higher in AN women than in US white women during the 1970s to early 1980s, but, through increased screening and treatment activities, rates have since decreased to be similar to the overall US average (1). HPV vaccines offer new opportunities for primary prevention of these cancers; both the bivalent HPV vaccine (Cervarix, GSK) and the quadrivalent HPV vaccine (Gardasil, Merck) are licensed and recommended for routine use in 11- or 12-year-old girls for the prevention of cervical cancer (2).

Infection with HPV causes virtually all cervical squamous cell carcinomas and cervical adenocarcinomas (3–5). Studies of invasive cervical cancers demonstrate that up to 99% of cervical cancer specimens have HPV detected (4–7). Of approximately 40 HPV types identified in the genital tract, at least 15 are considered carcinogenic (7). In the United States and worldwide, more than two-thirds of cervical cancers are due to HPV 16 and 18 (6, 8). While studies have evaluated different regions and countries, few have described genotypes in specific populations. A prior study of cervical cancer specimens from 1980 to 1989 found that HPV 16 was the most prevalent genotype in AN women: prevalence of HPV 16 was 77% in AN, 81% in Greenland Natives and 70% in Danish whites (9). In this study, 43% of HPV-positive AN specimens contained multiple HPV genotypes compared to 4% in Greenland Natives and 8% in Danish whites. HPV 31 and 33 were more frequently detected in AN specimens (21 and 30%, respectively) than in specimens from Greenland Native (3% each HPV 31 and 33) or Danish white (0% HPV 31 and 6% HPV 33).

Our study assessed HPV genotypes found in invasive cervical cancer specimens from AN women diagnosed from 1980 to 2007. The purpose was to characterize the preventable burden of HPV-associated cervical cancers in AN women to assess the potential impact of HPV vaccination on cervical cancer in this population.

## Methods

Invasive cervical cancer diagnosed from 1980 to 2007 in AN women living in Alaska aged 18 years and older were identified through the Alaska Native Tumor Registry (10). One representative formalin-fixed, paraffin-embedded block of the primary tumor was selected from each case at the Alaska Native Medical Center (ANMC). Serial sections were cut using precautions to prevent HPV contamination between blocks. The first and last sections were stained with hematoxylin and eosin, and they were reviewed by a study pathologist to confirm the histology. An intervening 10-micron section was extracted using the previously described high-temperature xylene-free method yielding a 100-µL extract (11). All extracts were tested with the Linear Array HPV Genotyping Test (Roche Diagnostics), which detects 37 HPV types (6, 11, 16, 18, 26, 31, 33, 35, 39, 40, 42, 45, 51, XR(52), 53, 54, 55, 56, 58, 59, 61, 62, 64, 66, 67, 68, 69, 70, 71, 72, 73, 81, 82, 83, 84, 89, IS39) and includes β-globin as an endogenous control. Samples with negative or inadequate (HPV and β-globin negative) results were re-tested with the INNO-LiPA HPV Genotyping Assay (Innogenetics). LiPA detects 29 HPV types (6, 11, 16, 18, 26, 31, 33, 35, 39, 40, 43, 44, 45, 51, 52, 53, 54, 56, 58, 59, 66, 68, 69, 70, 71, 73, 74, 81, 82) and includes HLA-DPB1 as an endogenous control. Inadequate samples (HPV negative and endogenous control negative) in both assays were excluded from this analysis. Fifteen HPV genotypes 16, 18, 31, 33, 35, 39, 45, 51, 52, 56, 58, 59, 68, 73 and 82 are considered oncogenic in this analysis (7). The protocol was approved by the Alaska Area IRB, Southcentral Foundation Scientific Review Board and the Alaska Native Tribal Health Review Board.

## Results

From 1980 to 2007, a total of 139 AN women were diagnosed with invasive cervical cancer while a resident of Alaska. Among these, a total of 136 were ≥18 years of age and 90 were diagnosed at the ANMC in Anchorage, Alaska. Archived tissues were located for 71 (79%) of ANMC cases >18 years and 62 (69%) contained representative cervical tumor.

The median age of the 62 women with an archived specimen available was 41 years, the same as the median age for all AN women diagnosed with invasive cervical cancer during the period 1980–2007 (Table I). Among women diagnosed during years 1984–2007, for which SEER Summary Stage was available, stage at diagnosis was similar (local, regional, distant and unknown stages) to all AN women diagnosed with invasive cervical cancer. Year of diagnosis was similar except for years 1985–1989 when proportionately fewer specimens were available. The majority of specimens were for women from Anchorage (47%) and women from the Yukon-Kuskokwim area (24%) of Alaska who presented or were referred to the Anchorage facility than for women living in other areas of Alaska.

**Table I:** Age, ethnicity, tumor stage, diagnosis year and geographic distribution of Alaska Native women with invasive cervical cancer, and Alaska Native women with cervical cancer specimens available, 1980–2007

Age range (years)	16–89	23–80
Mean	42.5	44.4
Median	41	41
Ethnicity (n=62)	%	%
Inupiaq/Yupik	43.9	54.8
Athabascan	44.6	30.6
Aleut	11.5	14.5
SEER summary stage	%	%
Local	58.7	52.0
Regional	28.4	36.0
Distant	10.1	12.0
Unknown	2.7	0
Year of diagnosis	%	%
1980–1984	25.5	24.3
1985–1989	21.9	11.2
1990–1994	16.3	17.7
1995–1999	14.2	17.7
2000–2004	9.9	12.9
2005–2007*	12.0	16.1
Geographic region (IHS Service Unit)	%	%
Anchorage	34.0	46.8
Fairbanks	15.6	3.2
Yukon-Kuskokwim	16.3	24.2
Southeast	10.6	4.8
Kotzebue	7.1	4.8
Ketchikan	5.7	3.2
Norton Sound	5.7	4.8
Arctic Slope	2.8	3.2
Bristol Bay	2.1	4.8

All 62 tissue specimens available for HPV typing yielded adequate results: 57 (91.9%) had HPV detected; 56 (90.3%) specimens were positive for one or more HPV types considered oncogenic (Table II). HPV 16 was detected in half of all specimens (31/62, 50%) and HPV 16 or 18 were detected in 61.3% (38/62) of specimens. HPV 16 was detected as a single infection in 41.9% (26/62). Eighteen (29%) specimens had oncogenic HPV other than HPV 16 and 18. One (1.6%) specimen had only HPV 69 detected. Two HPV genotypes were detected in 12.9% of specimens, and no specimens had more than 2 HPV genotypes detected.

**Table II:** Number and percentage of HPV genotypes detected in invasive cervical cancer specimens, Alaska Native women, 1980–2007 (N=62)

HPV type	Number	%
16	26	41.9
18	6	9.6
16+6^a^	1	1.6
16+18	2	3.2
16+33	1	1.6
16+54^a^	1	1.6
18+68	1	1.6
31	1	1.6
33+68	1	1.6
33	3	4.8
39+59	1	1.6
45	3	4.8
58	1	1.6
59	5	8.0
73	2	3.2
82	1	1.6
69^b^	1	1.6
Total	57^c^	91.9

a Low-risk HPV type.

b Non-oncogenic HPV type.

c HPV was not detected in 5 specimens.

The histologic types identified from 62 specimens were: squamous cell carcinoma (84%), adenocarcinoma (11%), and carcinoma, not otherwise specified (5%). Among the squamous cell carcinomas, HPV 16 was detected in 48% and HPV 18 in 12%. Among the adenocarcinomas, HPV 16 was detected in 29% and HPV 18 in 29%.

## Discussion

This study of cervical cancers in AN women found that 61% of tumors had evidence of HPV 16 or HPV 18. This is similar to findings from other studies on US women, in which approximately 70% of tumors were attributed to HPV 16 or 18 (2, 7). An HPV vaccine targeting oncogenic genotypes 16 and 18 could reduce cervical cancers in AN women by nearly two-thirds. If there is protection against other vaccine-related oncogenic types, through cross-protection or through new formulations of vaccine with different virus-like particles, vaccination could result in further reductions of cervical cancer (12, 13). It is important to note, one-third of HPV genotypes in our specimen population contained other oncogenic non-HPV 16 or non-HPV 18 genotypes alone (29%); types not targeted by the current vaccines. Regular cervical cancer screening for women at least 21 years or older is currently recommended for all women regardless of vaccination status.

Our study found some differences in HPV types in cervical cancer compared to the only prior study in AN women. In the prior study of cervical cancer specimens from AN women diagnosed from 1980 to 1989, 53 formalin-fixed and paraffin-embedded cervical cancer biopsies were assayed by PCR for 6 HPV genotypes (16, 18, 31, 33, 35 and 45) (9); overall, 98% were HPV positive. A higher percentage of HPV 16 alone (79% vs. 50%) and a lower percentage of HPV 18 alone were found in the prior study compared to this study (4% vs. 15%). The second most common HPV genotype in the earlier Alaska study was HPV 33 (30%); in this study, only 5 cases (8%) contained HPV 33. Multiple HPV genotypes were detected in 36.5% of cancer specimens in the earlier study, while this study found multiple genotypes in only 12.9% of tissues. Our study used different methods to reduce sample-to-sample viral carryover during thin-section preparation and different DNA extraction methods that most likely account for these differences. Twelve cases that were used in the prior study were retested in this study, although they were not necessarily the same tissue block. Of these 12, 9 (75%) were concordant for HPV genotype. In 2 of the 3 discordant cases, HPV genotype 16 was found by both methods, but the prior study detected additional HPV types not found using the current methods. The third discordant case revealed genotype 33 using the current methods versus genotype 16 in the prior study. Thus, in cases tested in both studies, the finding of a high percentage of multiple HPV genotypes was not corroborated using the current methods. One limitation of this study is that samples were selected based on the availability at one hospital pathology laboratory and may not be representative of all AN women with cervical cancer. Of the 90 individuals who received a diagnosis at ANMC, only 62 (69%) individuals had a cervical cancer specimen available. Possible reasons for unavailable tissue blocks include loss of specimen due to errors in labeling or misfiling in trays in the pathology laboratory, the storage box unlocatable in archives, or the cervical cancer specimen was completely used up in prior study. It is also important to note the classification of high-risk types depends on the study and methods. Munoz et al. classified 15 types as high risk, or oncogenic, and another three as possible oncogenic, types 26, 53 and 66 (7).

Studies in which HPV genotypes are detected in invasive cervical cancer provide important information to understand the impact HPV vaccines might have on reducing cervical cancer in defined populations. To date, most studies of different regions/ethnicities find similar proportions of cancers caused by HPV 16 or 18. Our study on AN women found a lower prevalence due to HPV 16 or 18, but given smaller numbers this is consistent with other studies. Evaluations of cancers due to HPV 16 and 18 in specific populations as well as vaccine impact monitoring on cancer reduction is warranted. One-third of all cervical cancers are caused by HPV types not prevented by vaccine, and provide support for continued Pap screening.

## Conclusions

Overall, almost two-thirds (61.2%) of the archived cervical cancers had detectible HPV types 16 or 18, a finding similar to studies of US women. As expected, a proportion of cancers would not be prevented by the current vaccines. HPV vaccination and cervical cancer screening are important prevention strategies for AN women.
